# Heat-vision based drone surveillance augmented by deep learning for critical industrial monitoring

**DOI:** 10.1038/s41598-023-49589-x

**Published:** 2023-12-15

**Authors:** Do Yeong Lim, Ik Jae Jin, In Cheol Bang

**Affiliations:** Department of Nuclear Engineering, Ulsan National Institute of Science and Technology (UNIST), Ulsan, 44919 Republic of Korea

**Keywords:** Energy infrastructure, Mechanical engineering, Information technology

## Abstract

This study examines the application of drone-assisted infrared (IR) imaging with vision grayscale imaging and deep learning for enhanced abnormal detection in nuclear power plants. A scaled model, replicating the modern pressurized water reactor, facilitated the data collection for normal and abnormal conditions. A drone, equipped with dual vision and IR cameras, captured detailed operational imagery, crucial for detecting subtle anomalies within the plant's primary systems. Deep learning algorithms were deployed to interpret these images, aiming to identify component abnormals not easily discernible by traditional monitoring. The object detection model was trained to classify normal and abnormal component states within the facility, marked by color-coded bounding boxes for clarity. Models like YOLO and Mask R-CNN were evaluated for their precision in anomaly detection. Results indicated that the YOLO v8m model was particularly effective, showcasing high accuracy in both detecting and adapting to system anomalies, as validated by high mAP scores. The integration of drone technology with IR imaging and deep learning illustrates a significant stride toward automating abnormal detection in complex industrial environments, enhancing operational safety and efficiency. This approach has the potential to revolutionize real-time monitoring in safety–critical settings by providing a comprehensive, automated solution to abnormal detection.

## Introduction

In the field of power plant industries, particularly nuclear power plants, the swift and accurate detection of machinery anomalies is of utmost importance. Stable operational performance hinges on the proactive identification and rectification of faults before they escalate into major accidents. While current monitoring systems employ advanced tools to measure essential parameters like temperature and pressure, they often fall short in detecting minor irregularities, necessitating manual expert evaluations^1,2^.

Given the complexity and vastness of power plants, there's an increasing demand for more efficient fault detection methods. Traditional surveillance systems, which rely heavily on manual inspections or expert evaluations, are becoming increasingly inadequate. As data from these large structures grows, manual interpretation becomes more challenging and time-consuming. This has led to a push towards automating data interpretation, capitalizing on advancements in artificial intelligence and machine learning^3^.

At present, most surveillance systems mainly rely on data monitoring techniques or depend on expert evaluations and on-site manual inspections. This situation opens up a vast area for further improvements to enhance safety and reliability in real-time power plant monitoring. As the amount of data gathered from large structures increases, depending on experts for data interpretation becomes more time-consuming and strenuous^4^. This situation calls for a significant shift towards automating data interpretation and analysis, taking advantage of developments in artificial intelligence and machine learning.

In line with this requirement, our study introduces a remote power plant fault monitoring technology. This technology combines the capabilities of the drone using infrared temperature measuring methods with modern deep learning object detection^5–7^ systems. In recent years, there has been a notable rise in efforts to automate data interpretation. In particular, deep convolutional neural networks (CNNs)^8–10^ have become powerful tools for classifying images across a wide variety of domains, including pavement crack detection, nuclear power plant damage inspection, steel box girder crack identification, concrete crack detection, and nuclear fuel rod pattern^11–19^.

Infrared thermography, a prevalent technique across various industries, offers the advantage of non-contact, remote, and straightforward detection. This method adeptly translates subtle temperature variations of target objects into comprehensive 2D visual representations, granting a refined insight into the condition of diverse installations and machinery. Yet, the challenge lies in identifying minuscule temperature shifts, indicative of potential anomalies, as they often elude direct detection and remain imperceptible to the human eye. Furthermore, relying on 2D imagery for rule-based decision-making compounds the intricacy of pinpointing faults. Integrating IR imaging with deep learning not only amplifies the accuracy of fault detection but also provides an in-depth, component-specific analysis. Specifically, convolutional neural networks (CNNs) have risen to prominence as a formidable instrument for image categorization, revolutionizing the paradigm of industrial fault identification. Recent innovations have seen the advent of bespoke CNN algorithms for niche applications.

For instance, machinery's remaining useful life has been predicted using CNN-based feature extraction from IR thermal images^20^, and an unsupervised Adversarial Regressive Domain Adaptation was used to apply to real data^21^. In addition, to efficiently diagnose prevalent defects in photovoltaic powerhouses, IR thermal image based-VGG16 architecture was modified to accurately detect common defects in photovoltaic modules^22^. IR thermal images have also been studied to recognize people, vehicles, or specific objects of interest in the dark using object detection algorithms^23,24^. In particular, recently by combining the temperature, surface characteristics, and structure of an object through thermal imaging with the deep learning algorithm, accurate texture recognition makes it possible to recognize physical characteristics such as texture and depth of an object even in complete darkness^17^. As in recent literature, the IR thermal imaging combined with deep learning has been employed across diverse fields for monitoring specific features^25^ or detecting particular objects. In this study, we aim to leverage this approach for detecting abnormalities in power plants, which consist of various mechanical components.

While the integration of temperature distribution insights with deep learning presents a forward-thinking approach, the expansive scope of large-scale plants necessitates an automated mechanism. In response, this study suggests employing drones to ensure comprehensive mobility, acting as an alternative to traditional manual inspections. Considering the vast and complex structure of power plants, which encompass numerous diverse components dispersed over extensive regions, the combination of drone technology with infrared imaging and deep-learning-driven fault detection emerges as a highly effective methodology. Given their aerial advantage, drones provide superior mobility over other robotic solutions, streamlining regular assessments and permitting concurrent operations across different zones. For recent related works, in a study by Kakiuchi et al.^26^, an aerial ubiquitous display (AUD) drone equipped with an infrared camera and projector was developed for night-time security, addressing labor shortages in Japan, and demonstrated effective real-time monitoring and information projection with an overall success rate of 72.7% in detecting suspicious activities and assisting lost individuals. Zhou et al.^27^ developed YOLO-CIR, an infrared object detection algorithm that integrates YOLO with ConvNeXt, introducing data augmentation and preprocessing tailored for infrared images, and incorporating attention mechanisms to enhance target, resulting in superior detection speed and accuracy compared to other prevalent algorithms, with potential applications in medical and industrial fields. Hu et al.^28^. Enhanced the YOLOv7-tiny model for drone-based object detection by introducing aspect-ratio-based anchor box assignment and a hard sample mining loss function, achieving improved detection performance on both infrared and visible light images, while retaining the model’s lightweight nature for practical drone applications. Gallagher and Oughton^29^ demonstrated that fusing RGB with thermal Long Wave Infrared (LWIR) imagery enhances object detection performance from air-based platforms, with the blended RGB-LWIR model outperforming standalone RGB or LWIR methods, and Khan et al.^30^ explored the use of drones and deep neural network in assessing thermal losses in building envelopes. Wu et al.^31^ introduced the illumination fusion module, a local adaptive illumination-driven input-level fusion technique for infrared and visible object detection, which enhances scene illumination perception and addresses image alignment challenge.

Recent advancements in object detection using drones, cameras, and infrared (IR) technology have predominantly leveraged the object detection model, known for its top-notch accuracy and fast processing speed. In this study, we endeavor to develop a novel approach that amalgamates drones, IR, and grayscale vision, with the primary objective of detecting abnormalities in power plants based on the object detection model A hallmark of our methodology is the fusion of grayscale and infrared imaging techniques, which is expected to significantly amplify object detection capabilities by integrating the complex details captured through grayscale imaging with the temperature insights gained from infrared imaging. This collaborative integration not only highlights the strengths of each individual technology but also alleviates their inherent limitations, providing a more robust and reliable monitoring framework.

Therefore, this combined approach aims to provide a more detailed, comprehensive, and precise abnormal detection model, greatly advancing the existing capabilities of nuclear power plant monitoring systems. In this study, we were trying to figure out abnormals and their exact locations within the complicated machinery commonly found in power plants. Using a small-scale thermal hydraulic replica of the nuclear power plant^32^, we aim to fine-tune the proposed method. This will be done by rigorously evaluating the effectiveness of various deep learning algorithms, thereby setting the path for technological progress in abnormal detection and monitoring systems in the power plant field.

## Results

### Power plant experiment

**Figure 1:**
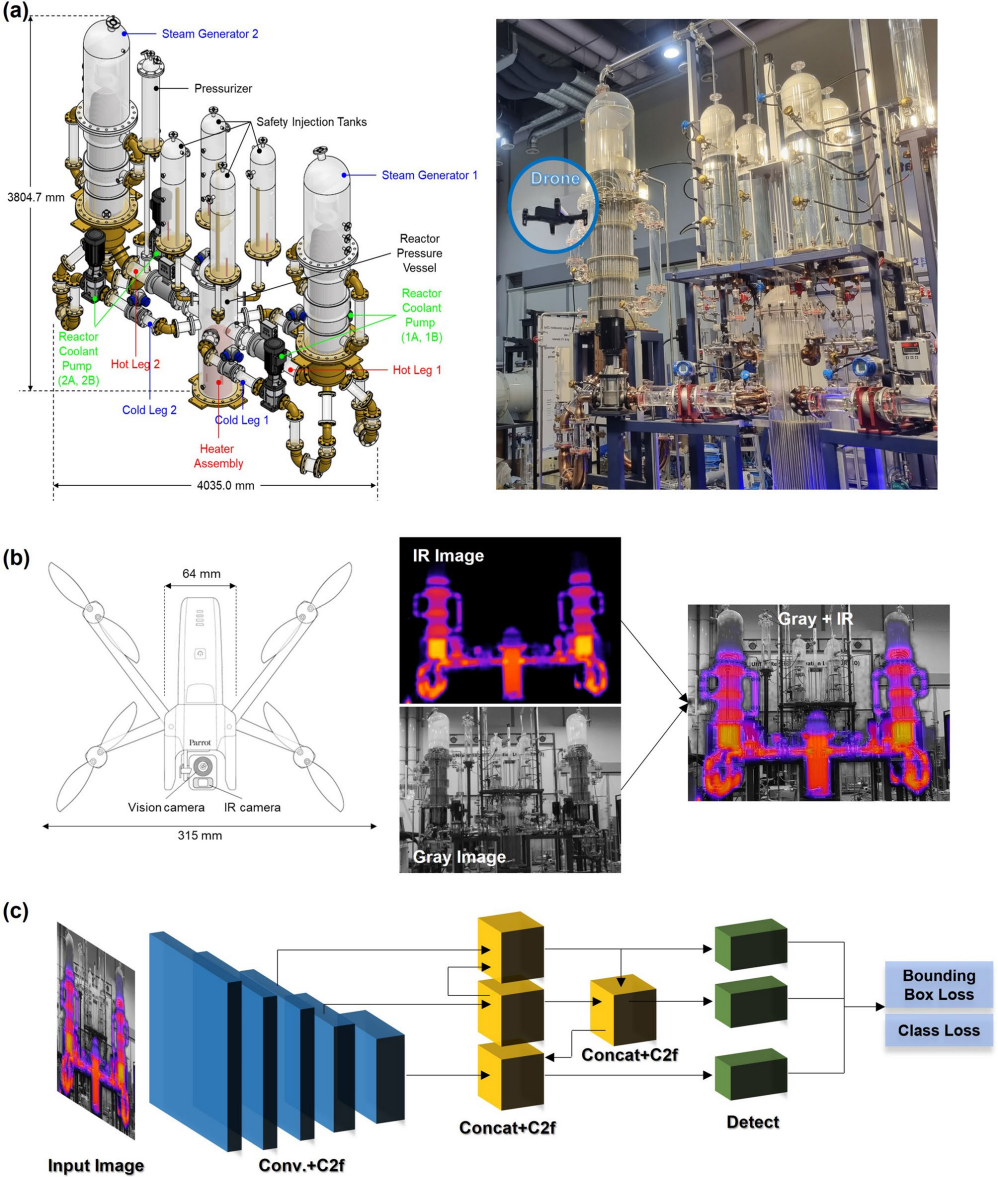
(**a**) Schematic representation of the scaled-down nuclear power plant experimental facility showcasing key components^18,32^ (left), and image of the actual experimental drone utilized at the URI-LO facility (right), (**b**) drone configuration^33^ and capture sample image data, and (**c**) deep learning method for object detection^5^.

To collect data on both normal and abnormal states in power plants, the experiments were conducted using the scaled-down nuclear power plant experimental facility named URI-LO^32^ as shown in Fig. 1a. This facility was meticulously designed to replicate the APR-1400 which is the modern pressurized water nuclear power reactor operational in Korea, featured substantially reduced volume, height, and diameter ratios of 1/1152, 1/8, and 1/12, respectively. The use of acrylic materials in its construction grants URI-LO transparency, providing an exceptional vantage point for observation and making it a critical asset for exploring the integration of advanced technologies in nuclear power plant environments. The primary function of URI-LO is to assess the feasibility of incorporating emerging technologies into nuclear power infrastructures, making it an invaluable resource for training, and evaluating deep learning techniques, particularly in the realms of infrared analysis and abnormal detection.

Integral to the experiments was a sophisticated drone system, equipped with both a vision camera and an IR camera, as illustrated in Fig. 1b. This dual-camera configuration facilitated the simultaneous capture of visual and thermal data, thus providing a detailed view of the facility's operations under various conditions. The role of the drone in capturing images was crucial, serving not only for real-time monitoring but also as a vital data source for deep learning applications. By utilizing this advanced imaging technology, a deeper understanding of the facility's behavior in both standard and exceptional transition scenarios was achieved. The deployment was aimed at enabling a comprehensive analysis and interpretation of the complex dynamics present in power plant operations.

The focus of the study was on identifying component abnormals within the primary system of the power plant. In URI-LO facility, the primary system consists of several elements, including a reactor pressure vessel (RPV), two steam generators (SG), four reactor coolant pumps (RCP), cold legs (CL), hot legs (HL), intermediate legs (IL), a pressurizer (PRZ), and a safety injection tank (SIT) as shown in Fig. 1a. The experiment was conducted by manually creating a abnormal state for some of the components and the normal operation state of the facility. The conditions for each experiment were as follows.

To simulate normal operating conditions, we aimed to mimic an insulated nuclear power plant by reducing the surface temperature on URI-LO, which is composed of acrylic material with low thermal conductivity. For this approach, the core power was set to 30 kW and the core flow rate was maintained at 6.6 kg/s, maintaining a maximum temperature threshold of 40 °C. As a result, the maximum temperature inside the hot legs was stabilized at 40 °C, with excess heat effectively dissipated through the steam generators.

Meanwhile, two types of abnormal state experiments were conducted to simulate critical scenarios in nuclear reactor operations. The first experiment involved reducing the water level inside the steam generator among the URI-LO components. This scenario was chosen because a decrease in the steam generator's water level can cause issues in removing heat generated from sources like the nuclear core, presenting a scenario that must be avoided in actual operations. For simulate this scenario, the experimental procedure was manually initiated by intentionally draining the fluid level in the steam generator at a rate of approximately 3 mm/s. The second experiment involved stopping the fluid circulation pump of the primary system. For this, tests were conducted where either two or all four of the operating pumps were simultaneously stopped during normal operation conditions. This was selected because fluid circulation and convective heat transfer are essential for heat removal; stopping the pump could significantly disrupt the heat balance. These fluid dynamics and potentially critical transitions were carefully monitored using drones equipped with both vision and IR sensors. This approach allowed for accurate data capture during unusual transient states, providing valuable insights for subsequent analysis. IR and grayscale images from the drone were combined, as shown in Fig. 1b, to identify abnormalities more effectively. This data initiated a deep learning process using the object detection deep learning model, as illustrated in Fig. 1c.

**Figure 2:**
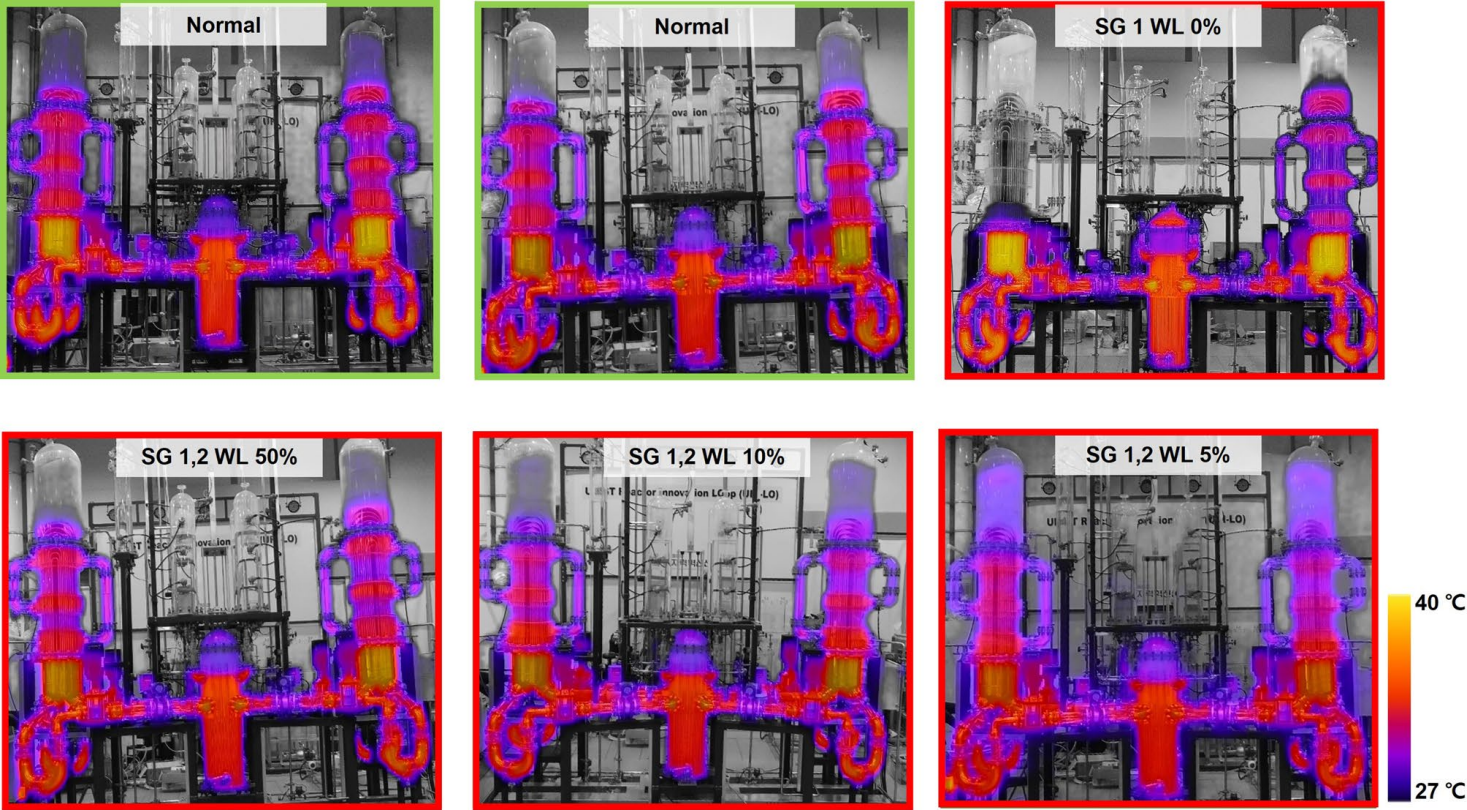
Sample images from drone IR and gray vision capture at normal and SG abnormal state.

As a result, gray vision and thermal combined images from the SG drain transient experiment are shown in Fig. 2, showing the normal state (Green Box) of the system and various abnormal state at water levels for SG 1 and 2 (50%, 10% and 5%, Red Box). The thermal characteristics captured by the drone's IR imaging system provide crucial insights into these states. In the case of SG 1 reaching a 0% water level, complete fluid evaporation was observed, leading to a significant reduction in heat transfer. This resulted in a noticeable temperature drop, falling below the thermal imaging range, and appearing gray in the imagery. This critical state, indicative of halted heat transmission, could signal operational issues or malfunctions.

In addition, during the instances where the water level ranged between 50 and 5%, the visual and thermal differences were not significantly discernible to the naked eye. This can be attributed to the residual fluid clinging to the SG walls and the ongoing heat transfer facilitated by the evaporated vapors, maintaining a relatively stable temperature profile. It delineates a scenario where despite the lowering levels of water, the system still manages to sustain a degree of thermal stability due to the residual heat and fluid dynamics at play. However, it was identified that in such transient situations, discerning issues visually from the overlaid IR and grayscale images captured by the drone became rather challenging. This underlines the necessity for more sophisticated analysis and monitoring tools that can accurately identify and signal deviations or potential issues in real-time, fostering a proactive approach to system management and maintenance.

**Figure 3:**
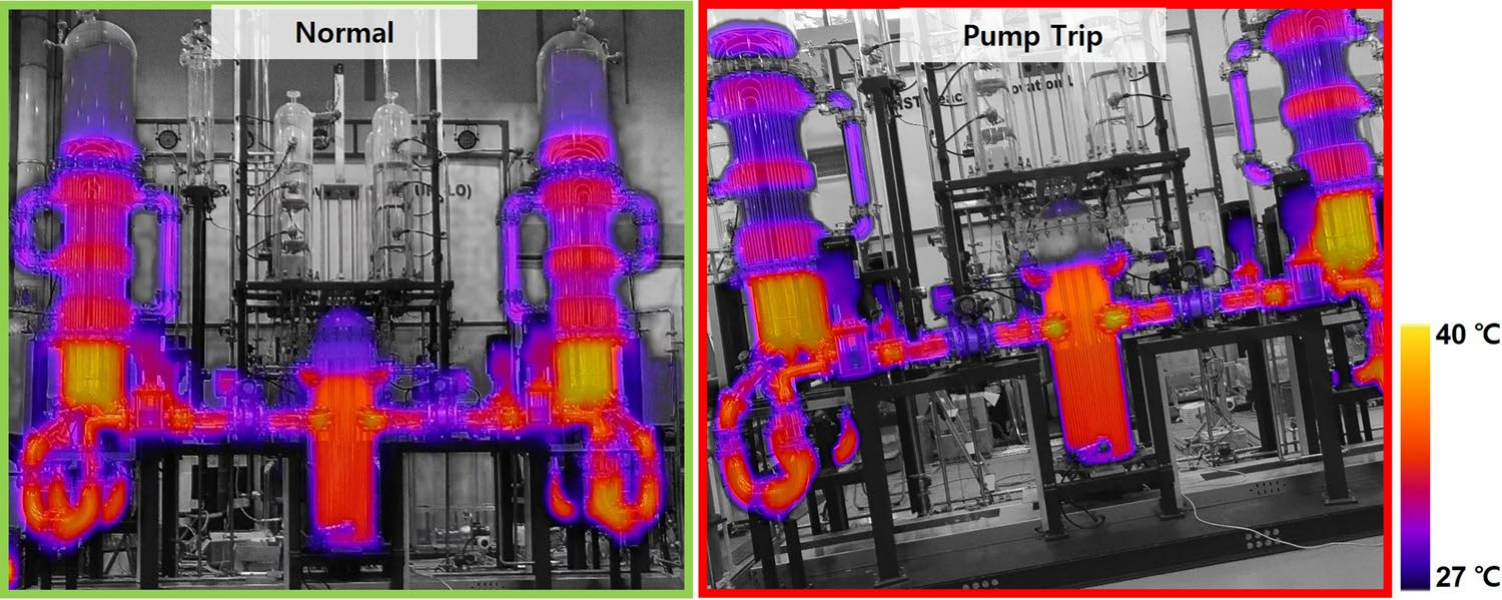
Sample images from drone IR and gray vision capture at normal and RCP abnormal state.

In the case of the fluid circulation pump stop experiment, 2–4 of the 4 pumps were stopped simultaneously in normal operation, and the drone filming results are shown in Fig. 3. These results are thermal image results taken during normal operation of the pump, when the left pump is stopped, and when the pumps on both sides are stopped. As in the previous experiment results, there appears to be a temperature change, but it is so minimal that it is very difficult to visually distinguish whether there is an abnormality or not. Therefore, the dataset of experimental results from the URI-LO facility were composed as the operating state in which all components were normal, one or both steam generators were abnormal, and two or four of the RCPs were abnormal.

### Optimal abnormal monitoring model

In the preceding section, we elucidated the intricacies involved in capturing thermal and grayscale imagery of the facility using drones, which exhibited significant potential in monitoring the abnormal states within the facility’s major components. The experimental results have demonstrated that while drones can effectively capture both visual and thermal images remotely, determining the state of the system from these images remains challenging due to the subtlety of changes. It was observed that even significant operational anomalies sometimes resulted in only minor visible or thermal variations, making it exceedingly difficult to discern system abnormalities through manual analysis. This underscores a critical limitation in relying solely on conventional imaging techniques for monitoring complex systems like those in nuclear power plants.

To address this challenge, as illustrated in Fig. 1c, the deep learning for object detection was conducted. This approach was chosen to enhance the capability to detect and interpret subtle changes in the system that are not easily identifiable by standard monitoring methods. By integrating deep learning algorithms, we aim to not just identify potential anomalies at an early stage but also gain predictive insights that could preemptively address risks associated with such transient states.

In this study, deep learning-based object detection, traditionally used for identifying instances of semantic objects (such as humans, buildings, or cars) in digital images and videos, was applied with a specific focus on classifying components of a power plant system. We divided the classes into 'normal component classes' (RPV, SG, RCP, PRZ, SIT, CL, HL, IL) and 'abnormal component classes' (SG-Fault, RCP-Fault). Normal classes were represented with green bounding boxes, while abnormal classes were highlighted with red bounding boxes. This color-coded bounding box approach was designed to facilitate easy identification of abnormal components. To achieve this, we modified representative object detection models, utilizing both one-stage (YOLO^34–36^, NanoDet^37^) and two-stage (Mask R-CNN^7^) object detection models. The aim was to evaluate the performance in detecting abnormalities and to identify the most optimal model configuration for this specific application.

To find the optimal model using various models, A total of 9 models were trained, using the nano, small, medium model of YOLO v5 and YOLO v8, NanoDet-Plus model, Mask R-CNN-Resnet101, and Mask R-CNN-Resnet50 model. Such model learning and comparison requires very accurate prediction performance of deep learning models in safety–critical industries such as nuclear power plants. Therefore, the criteria were set so that the model with the highest prediction performance was the optimal model regardless of the model size or number of parameters, and the mAP50 and mAP50-95 performance indicators were compared for quantitative comparison. Additionally, we compared the size of FLOPs with respect to the time it takes to predict a single image to understand the practical implications of using these models in real-time monitoring scenarios. In all training cases, if no improvement in validation loss was observed for 50 consecutive epochs, training was considered to have reached saturation and training was stopped.

Table 1 provides a comprehensive comparison of model prediction performance in object detection using deep learning, highlighting essential metrics such as mAP50, mAP50-95, Parameters (Params), and Floating-Point Operations Per Second (FLOPS). The analysis reveals that the YOLOv8 series, particularly the medium configuration (v8m), demonstrates exceptional performance with the highest mAP50-95 score of 0.804. This score is indicative of the model's superior capability to accurately detect objects across various classes and sizes, which is crucial for complex detection tasks. The progression in mAP scores from the YOLOv5 to YOLOv8 series underscores advancements in model architectures and algorithm efficiency.

**Table 1:** Model prediction performance comparison of object detection with deep learning.

Model	mAP50	mAP50-95	Params	FLOPS
YOLOv5n^35^	0.973	0.742	1.9 M	4.5 G
YOLOv5s^35^	0.970	0.767	7.5 M	16.5 G
YOLOv5m^35^	0.971	0.756	21.2 M	49 G
YOLOv8n^36^	0.977	0.779	3.2 M	8.7 G
YOLOv8s^36^	0.981	0.799	11.2 M	28.6 G
YOLOv8m^36^	0.981	0.804	25.9 M	78.9 G
NanoDet-Plus^37^	0.959	0.716	1.17 M	1.52 G
Mask R-CNN-101^7^	0.919	0.716	55 M	937 G
Mask R-CNN-50^7^	0.916	0.711	36 M	890 G

In contrast, the NanoDet-Plus and Mask R-CNN models present a different aspect of model performance. NanoDet-Plus, with the lowest FLOPS in the group, shows a decent mAP50-95 score of 0.716. This model's strength lies in its efficiency, making it suitable for scenarios where computational resources are constrained. On the other hand, the Mask R-CNN models, despite their significantly higher computational demand (evidenced by their high FLOPS), do not yield proportional improvements in mAP scores. Specifically, Mask R-CNN-101 and Mask R-CNN-50 record mAP50-95 scores of 0.716 and 0.711, respectively. This disparity highlights the importance of balancing computational complexity with performance gains.

Furthermore, the FLOPS metric is directly tied to inference time and, by extension, to the applicability of models in real-time scenarios. Higher FLOPS typically translate to longer inference times, as more computational operations are required. This can be a limiting factor in real-time applications where rapid processing is essential. For instance, while the YOLOv8 models exhibit significant improvements in detection accuracy, their higher FLOPS, such as 78.9 G for YOLOv8m, may result in extended inference times, potentially hindering their deployment in scenarios demanding quick responses.

Therefore, Table 1 not only showcases the advancements in deep learning models for object detection but also emphasizes the trade-offs between accuracy, computational complexity, and speed. It highlights the need for a careful selection of models based on the specific requirements of the application. While models like YOLOv8 excel in accuracy and are suited for complex detection tasks, models like NanoDet-Plus offer efficient alternatives for constrained environments. Similarly, while higher FLOPS can lead to better accuracy, they also necessitate consideration of the impact on inference time, especially for real-time applications. This comprehensive analysis aids in making informed decisions to achieve an optimal balance between these critical factors.

**Figure 4:**
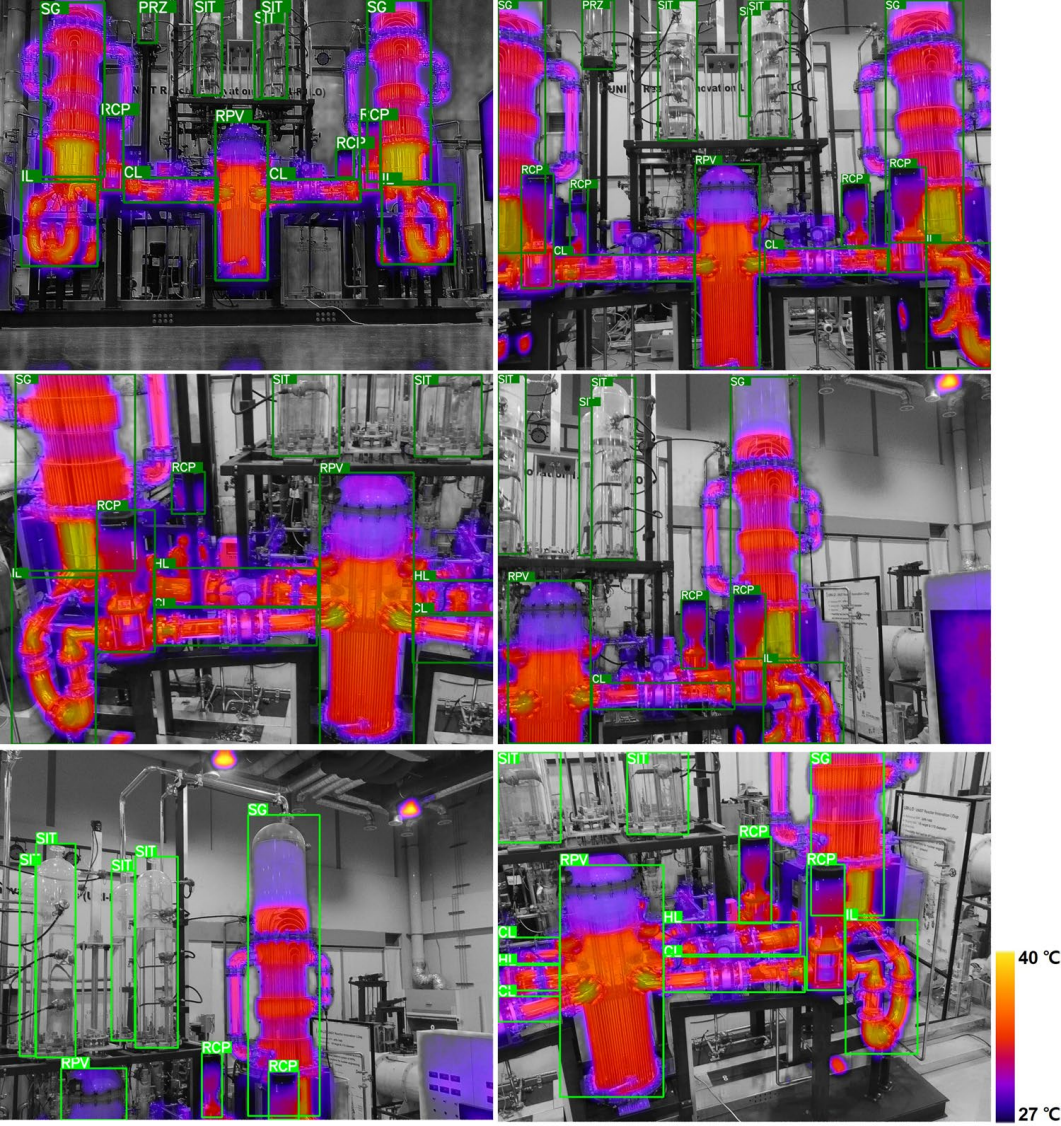
Drone–Gray-IR based multi-component object detection at normal operation state.

In critical industrial areas where safety and reliability are paramount, prioritizing the accuracy of deep learning models is essential. Therefore, this study focused on the model with the highest average average precision (mAP50-95) index, and we selected the YOLO v8m model for image testing. Our results are illustrated in Fig. 4, which shows results derived from object detection under normal operating conditions.

The YOLO v8m model was observed to be adept at recognizing various components of the facility when the drone approaches from the front. This high level of capability can be maintained even if the drone adjusts altitude or moves laterally during operation. This highlights the model's ability to maintain a high level of detection accuracy across a variety of operational scenarios and demonstrates its robustness and reliability for real-world applications.

**Figure 5:**
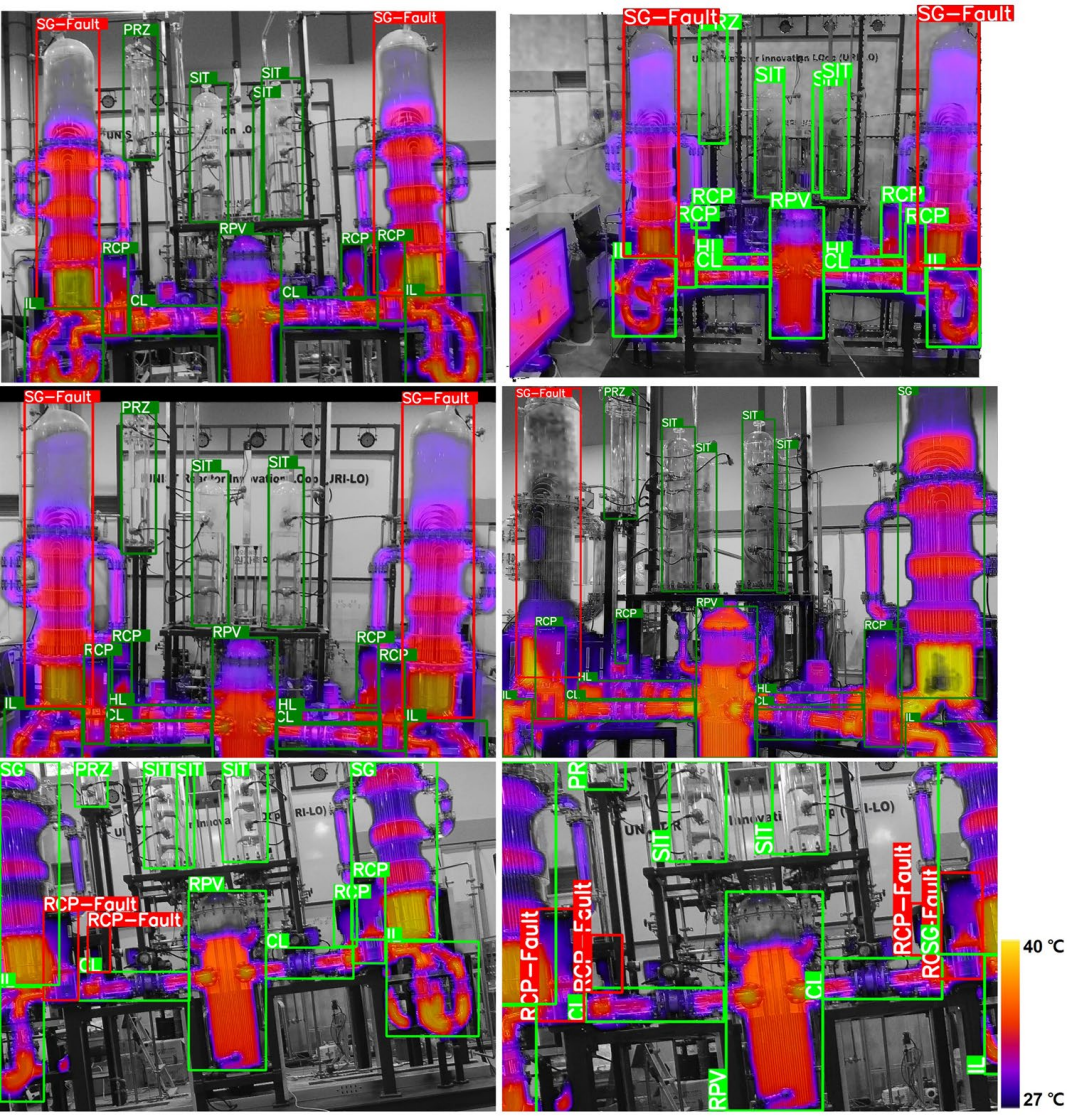
Drone–Gray-IR based multi-component and abnormal detection.

In contrast, Fig. 5 shows model testing results for images depicting anomalous states. By capturing the subtle temperature changes that occur due to fluid drain inside the steam generator, the YOLO model accurately captured the SG's fault. Likewise, the stoppage of the pump was accurately identified. The analysis revealed the model's ability to accurately detect and adapt to anomalies, particularly identifying changes in water level (WL) in the steam generator (SG) at different stages of the drainage process. This demonstrates the adaptability and accuracy of the model in identifying deviations from standards, highlighting its potential as a valuable tool for monitoring anomalies in critical industrial environments.

## Discussion

**Figure 6:**
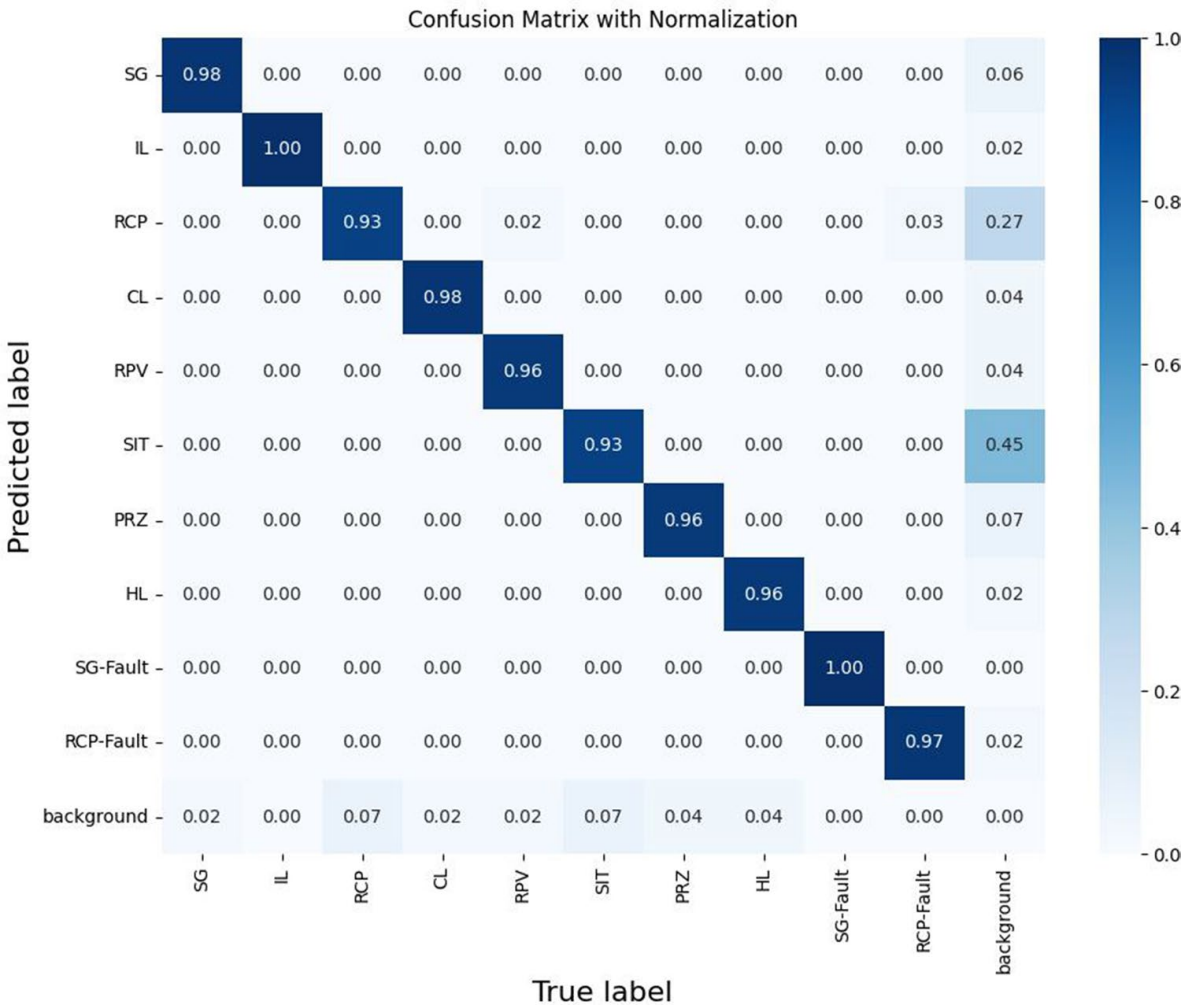
Normalized confusion matrix of object detection.

To examine the detection performance in more detail, we analyzed the results through a confusion matrix as shown in Fig. 6. Here, it was confirmed that most components, including RPC, SG, SG abnormality, CL, HL, and IL, were accurately detected. Upon classification, there were cases of misclassification regarding RCP and SIT. In particular, RCP and SIT were sometimes misclassified as IL and RPV, respectively. The underlying reason for this discrepancy may be the morphological similarity between SIT and RPV, which are cylindrical, which could lead to incorrect identification. Likewise, misidentifications between RPC and IL sometimes seem to stem from complex shapes that can confuse the model.

Additionally, it was pointed out that various objects in the background, such as monitors, lights, and pipes, were incorrectly identified as multiple components. This problem mainly occurred because these elements were not explicitly trained during the learning phase, resulting in a high misidentification rate. We identify that this problem can be addressed through a more detailed labeling and learning process that specifically targets these objects, which can help improve the overall accuracy of the system.

Therefore, this study highlights the promising potential of integrating drone technology with IR gray-scale vision image capture and deep learning-based object detection methodologies. Especially in environments such as nuclear power plants, where heat generation is high, numerous components are integrated, and safety is paramount, this approach paves the way for real-time remote monitoring capabilities. By further fine-tuning the process, we expect to develop a robust system that can significantly improve the safety protocols of these critical facilities and represent a new frontier in leveraging technology to improve safety.

This research sets out on a path to innovatively combine drone technology with infrared (IR) grayscale vision imaging and deep learning algorithms, with the goal of pioneering new real-time remote monitoring systems, especially those relevant to high-risk environments such as nuclear power plants. These plants are complex combinations of components that generate significant heat, so safety is a top priority.

Our analysis highlighted the ability of the YOLO v8m model to identify anomalies with improved accuracy, as illustrated in the results presented in Figs. 4 and 5. It has shown remarkable adaptability in skillfully recognizing various components under normal operating conditions and identifying abnormalities under abnormal conditions. We highlight its potential efficacy as a pivotal tool in abnormal monitoring procedures.

Nonetheless, the study also revealed certain shortcomings, particularly misclassification of complex-shaped components and objects in the background, pinpointing areas requiring further improvement. Using a more granular approach in the labeling and training phase can potentially ameliorate these issues, improving accuracy and reliability.

In conclusion, it is clear that the combination of drone technology and deep learning-based object detection methodologies offers a promising way to enhance safety protocols in critical facilities. By fostering progress in this direction, we hope to pave the way for deploying powerful systems that act as a bulwark of safety assurance, leveraging technology to emphasize safety measures in critical installations. The preliminary results presented in this study serve as a precursor to the potential progress that can be made in this area and call for further research and development to fully realize these integrated capabilities in creating a safer operating environment.

## Methods

### Experimental facility and drone monitoring system

For the remote monitoring and capturing of visuals and thermal images, we used a drone named the Parrot Anafi Thermal^33^, which comes with both IR and vision sensors. This setup allows for simultaneous vision and IR image capture. This dual system is vital for creating a detailed database that combines both kinds of images, improving the accuracy and reliability of detecting component abnormals. The IR sensor we used was the FLIR Lepton 3.5, which is well-regarded for its ability to capture and send data within a set range, becoming a key tool in identifying component temperature issues. At the same time, the drone's built-in vision camera helps in pinpointing the exact locations of the components being analyzed. This cooperative approach, which uses both normal and thermal images, forms the base for a strong database, marking a significant step forward in the field of nuclear power plant monitoring and maintenance.

### Object detection with deep learning

Recently, the role of deep learning methods in detecting objects has seen a substantial increase. This surge is largely due to their high accuracy and ability to process data in real time, which is especially important in the nuclear power sector where quick and accurate monitoring of component conditions is vital. To enhance this process, drones equipped with advanced IR and vision sensors are employed. These sensors, coupled with grayscale imaging, can potentially amplify the object detection capabilities. Grayscale images help in detailed object detection as they represent varying light intensities in different portions of an image with distinct shades of gray, sometimes providing better detail compared to colored images.

**Figure 7:**
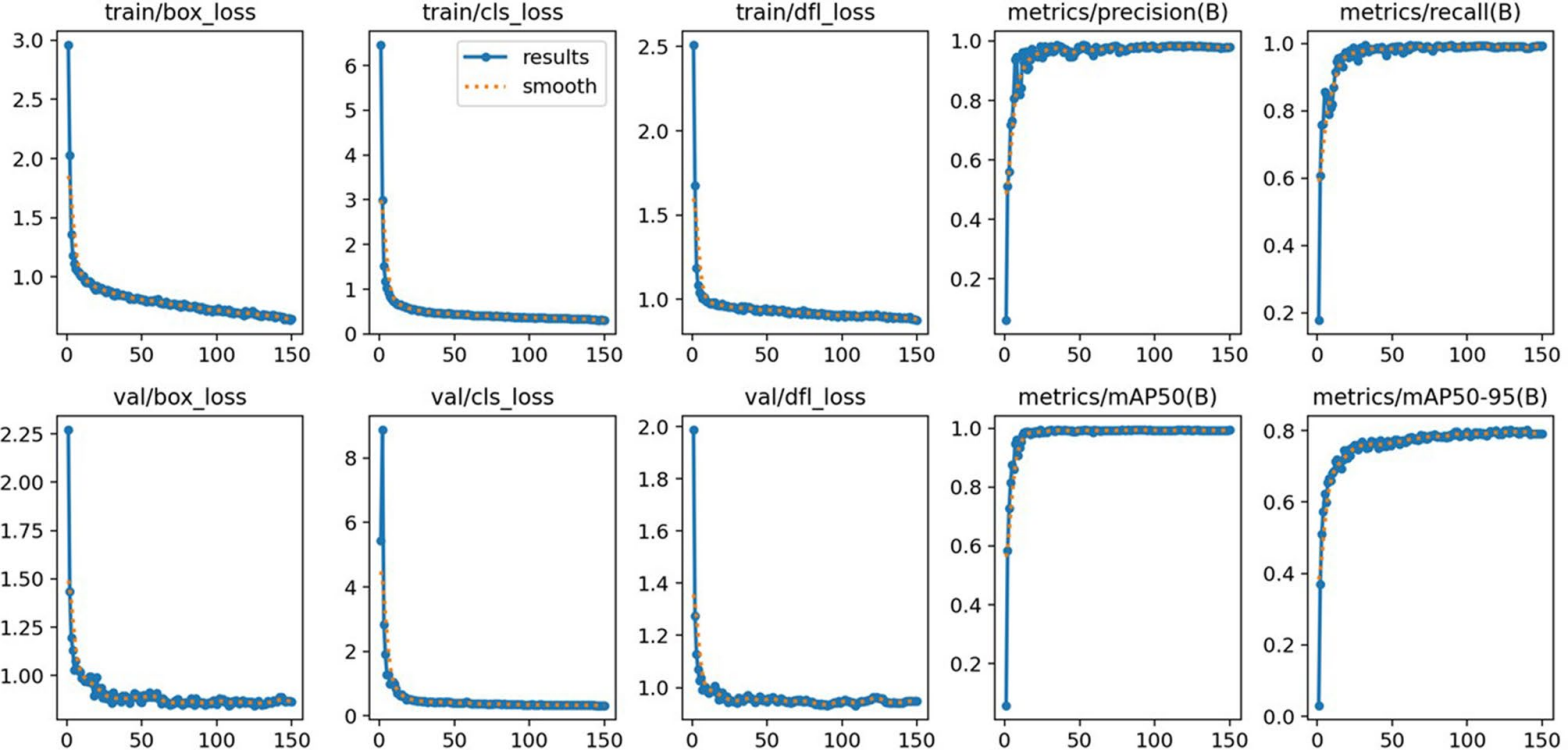
Loss function and prediction evaluation results for YOLOv8m model at each iteration.

YOLO^34–36^: as shown in Fig. 1c, The YOLO model is a one-stage object detection model, and its architecture uses a single Convolutional Neural Network (CNN) that processes the entire image at once. This splits the image into a grid, with each grid cell predicting a number of bounding boxes and a confidence score for those boxes. At the same time, YOLO predicts the probability of the object class within that box. The model applies two specific loss functions during training. One is for the accuracy of bounding box coordinates and the other is for the accuracy of class prediction. This architecture allows YOLO to quickly detect objects and their classifications in a single evaluation, making it highly efficient for real-time applications. And sample results of the YOLO training was depicted in Fig. 7, showing converged training outcomes for hundred epochs.

NanoDet-Plus^37^: NanoDet-Plus is an improved version of the original lightweight NanoDet, an anchorless object detection model that is efficient and effective for real-time applications. We introduce an innovative label assignment strategy that includes a simple guidance module and a dynamic label assigner to help optimize training for small models. NanoDet-Plus also features an enhanced light function pyramid network called Ghost-PAN, which enhances the convergence of multi-layer functions.

Mask R-CNN^7^: Mask R-CNN, an evolution of Faster R-CNN, is a sophisticated model for object detection that uniquely integrates region proposal with convolutional neural networks. Utilizing a Region Proposal Network (RPN) to pinpoint object locations, it employs RoIAlign to meticulously align the extracted features with the input, ensuring precise localization. The model simultaneously predicts class labels and bounding box offsets for each RoI, effectively distinguishing and localizing multiple objects within an image. This dual-output approach enables Mask R-CNN to not only detect objects with high accuracy but also segment them, providing detailed information on the object's shape and position. Its versatility and precision have made it a go-to framework for complex object detection tasks in various domains.

Setting up clear performance measurement standards is vital. These standards include metrics like mAp50, and mAp50-95 score. Here's a simple breakdown of these metrics: mAp50 and mAp50-95 score—these metrics are used to evaluate the performance of object detection models, with the numbers indicating the IoU (Intersection over Union) thresholds. The higher the threshold, the more stringent the evaluation.

**Figure 8:**
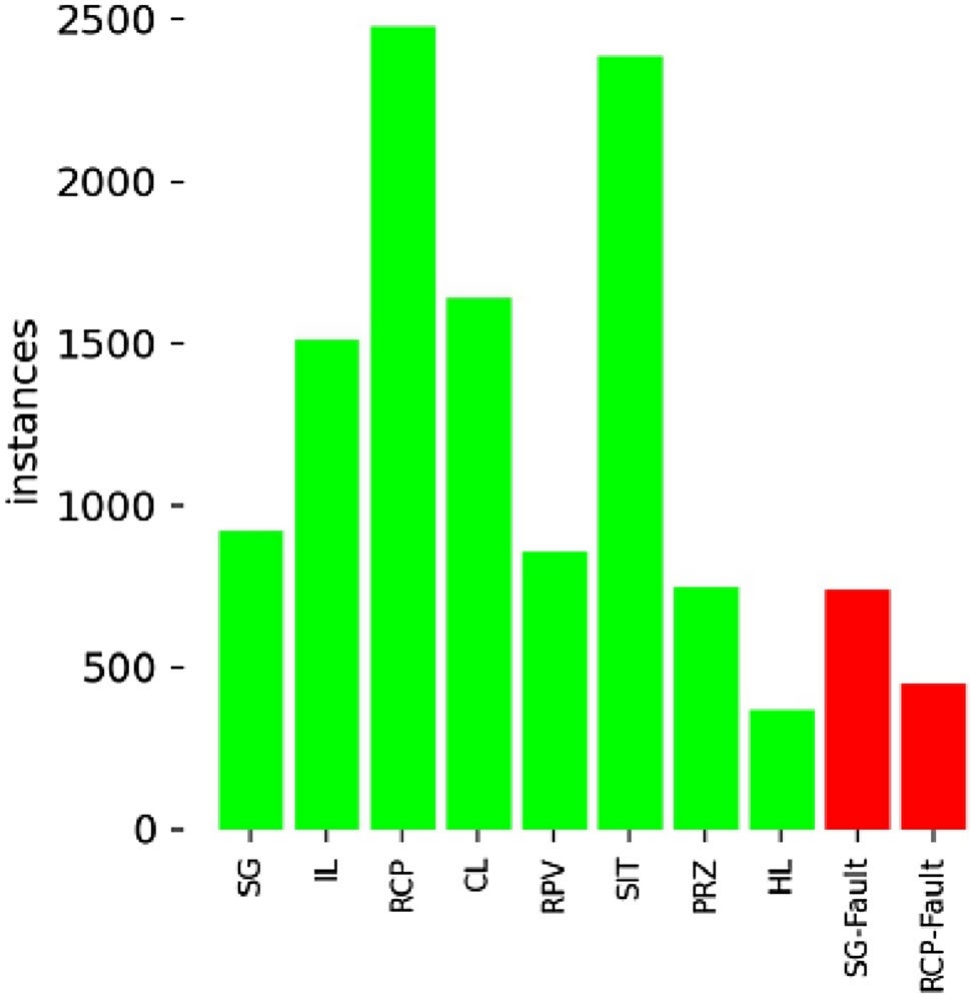
Training dataset configuration.

The object detection model in this study was trained on dataset comprising 1386 images, categorized into 10 unique classes based on specific labeling criteria. Within this dataset, a substantial portion, totaling 1038 images, was designated for capturing abnormal states. These images are marked with the label 'Fault', indicating the presence of at least one fault component within them. The remaining 348 images in the dataset depict normal operational states of the components. Each image is notable for capturing multiple components, thereby showcasing a variety of classes that fall within the camera’s observational range, therefore the comprehensive distribution of class instances was shown in Fig. 8.

In order to improve the accuracy of the predictions, specific modifications were applied during the testing phase. These included adjusting the size of the input data and fine-tuning the learning rate to optimize performance. The dataset was randomly divided for training, validation, and testing purposes, with 969 images (70%) used for training, 138 images (10%) for validation, and 279 images (20%) for testing. It is essential to highlight that all learning and testing processes were conducted in a Python environment using PyTorch (YOLO, NanoDet) and TensorFlow (Mask R-CNN) frameworks, with the computational support of an RTX 3090 GPU. This hardware setup ensured efficient handling of the dataset and the deep learning tasks.

## Data Availability

The dataset collected from the plant experimental apparatus, along with the associated code used in this study, can be accessed on GitHub at https://github.com/TH-dyLIM/Plant-Abnml-DroneIR-Detection.git. An inference video has also been provided within the repository for further clarity.
